# Neurophysiological Basis of Short-Chain Fatty Acid Action in Pain Modulation: Therapeutic Implications

**DOI:** 10.3390/ijms26168082

**Published:** 2025-08-21

**Authors:** Mamoru Takeda, Yukito Sashide, Syogo Utugi

**Affiliations:** Laboratory of Food and Physiological Sciences, Department of Life and Food Sciences, School of Life and Environment Sciences, Azabu University, 1-17-71, Fuchinobe, Chuo-ku, Sagamihara 252-5201, Japan; sashide0112@hotmail.co.jp (Y.S.); f22015@azabi-u.ac.jp (S.U.)

**Keywords:** gut microbiota, short-chain fatty acids, neurophysiology, nociceptive pain, inflammatory pain, anesthesia, non-steroidal anti-inflammatory drugs

## Abstract

The gut microbiota influences both energy metabolism and central nervous system (CNS) functions. This influence is mediated by humoral factors, including various metabolites, neurotransmitters, cytokines, and hormones, in addition to neural pathways such as the vagus nerve. Notably, short-chain fatty acids (SCFAs)—comprising acetic, propionic, and butyric acids—merit specific attention. These compounds originate from the anaerobic fermentation of dietary fibers by the gut microbiota. Growing evidence indicates that SCFAs confer beneficial effects on diverse pain conditions. Although previous review articles have summarized animal studies suggesting the possibility that SCFAs can alleviate pathological pain, there are few reviews on the neurophysiological mechanisms by which SCFAs modulate the excitability of nociceptive neurons in the pain pathway under nociceptive and pathological conditions. Extending previous in vitro findings, our laboratory recently conducted in vivo neurophysiological studies using animal models to explore the pain-relieving properties of SCFAs. Our published results demonstrate two significant effects: (i) an intravenous anesthetic action against nociceptive pain and (ii) an anti-inflammatory contribution to chronic pain alleviation. This review synthesizes the current understanding of the mechanisms by which SCFAs modulate pain and explores their contribution to the attenuation of nociceptive and/or pathological pain. Furthermore, we discuss their prospective clinical application

## 1. Introduction

Beyond its role in the digestive system, recent findings indicate that the gut microbiota influences the maintenance of biological functions via bidirectional communication with the central nervous system (CNS). Moreover, studies have linked it to the pathogenesis of various diseases [1]. The gut microbiota influences energy metabolism and CNS functions via a combination of humoral factors, including metabolites, neurotransmitters, cytokines, and hormones, and neural pathways, exemplified by the vagus nerve [1,2,3,4]. Of particular interest are short-chain fatty acids (SCFAs), such as acetic, propionic, and butyric acids, which the gut microbiota produces via the anaerobic fermentation of dietary fibers [3,4].

SCFAs have been shown in previous research to bind to G-protein-coupled receptor 41 (GPR41)/free fatty acid receptor 3 (FFAR3), GPR43 (FFAR4), and GPR109 (hydroxy-carboxylic acid receptor: HCAR2) on the cell membrane [5]. This binding subsequently activates various intracellular signal transduction systems, through which SCFAs exert their physiological functions [6]. SCFAs also influence blood–brain barrier (BBB) permeability along with the function of neurons and glial cells [1]. Consequently, substantial preclinical and clinical findings indicate a potential role for SCFAs across a range of neurological conditions: anorexia nervosa, Parkinson’s disease, Alzheimer’s disease [7], autism spectrum disorder [7,8,9,10,11], and chronic pain [4,12].

Compared to specific pathogen-free (SPF) mice, germ-free (GF) mice exhibit significantly reduced levels of the three most abundant SCFAs (acetic, propionic, and butyric acids) [13]. Prior research indicates that germ-free (GF) mice manifest visceral hypersensitivity [14] and prolonged migraine-like pain in the nitroglycerin (NTG) model [15]. These pain behaviors are reversible, as demonstrated by their normalization following fecal microbiota transplantation (FMT) [14,15]. Moreover, germ-free (GF) mice exhibit increased levels of pain-related receptors and cytokines in their spinal cord [14]. Furthermore, in GF rats that underwent FMT using feces from an irritable bowel syndrome (IBS) patient, Chinese herb (Coptis chinensis) and berberine treatment elevated acetate, propionate, and total SCFA concentrations, alongside enriching various SCFA-producing bacteria, which subsequently inhibited visceral hypersensitivity [16]. Collectively, these findings indicate that SCFAs may play a role in modulating pain hypersensitivity. Furthermore, accumulating evidence demonstrates that SCFAs exert beneficial effects across diverse pain conditions [12,17,18].

Previous review articles have summarized animal studies indicating the potential for SCFAs to alleviate pathological pain [19,20]. However, there remains a scarcity of reviews detailing the neurophysiological mechanisms through which SCFAs modulate the excitability of nociceptive neurons in the pain pathway across both nociceptive and pathological conditions. Building on prior in vitro experimental findings, our laboratory recently investigated the pain-relieving effects of SCFAs using an in vivo neurophysiological approach in animal models. Our published studies highlight two key effects: (i) an intravenous anesthetic effect on nociceptive pain and (ii) an anti-inflammatory effect contributing to chronic pain relief. This review synthesizes the current understanding of the mechanisms by which SCFAs modulate pain and explores their contribution to the attenuation of nociceptive and/or pathological pain. Furthermore, we discuss their prospective clinical applications.

## 2. Pain Classification

Serving as a warning signal, physiological pain is often termed nociceptive pain. Conversely, pathological pain denotes a state in which this inherent “biological warning signal” function has been lost. This is characterized by alterations in neurons within the pain transmission pathway and sustained signal transduction activation, which leads to chronic pain. This pain severely diminishes quality of life and often persists even after significant tissue damage has healed [21]. Pathological pain lacks a protective warning function and encompasses conditions such as inflammatory and neuropathic pain. Inflammatory pain results from the sensitization of nociceptors by inflammatory chemicals (e.g., prostaglandin E_2_ [PGE_2_]) at sites of tissue damage, for example in burns and joint pain. Neuropathic pain, conversely, stems from nerve damage and uniquely endures after the initial injury has been resolved. Clinical examples include diabetic neuropathy, sciatica, and trigeminal neuralgia, as well as cases observed in dentistry following procedures like extractions and implants. Common symptoms associated with these conditions include hyperalgesia (increased sensitivity to painful stimuli) and allodynia (pain from normally non-noxious stimuli) [22,23]. The prevailing hypothesis suggests that these pathological pain conditions stem from plastic changes within the sensory neurons of somatic sensory pathways, often precipitated by inflammation or injury to peripheral tissues [24]. Following tissue inflammation or injury, peripheral sensitization occurs due to the enhanced excitability of peripheral nerve endings and chemical communication (e.g., neuronal and neuro-glial crosstalk) in sensory ganglia. This process is commonly understood to cause central sensitization, which includes hyperalgesia [25,26,27,28].

## 3. Fundamentals of the Trigeminal Nociceptive Pathway

This review explores the potential role of short-chain fatty acids (SCFAs) in mitigating nociceptive and pathological pain. Building on our recent findings demonstrating SCFAs’ influence on trigeminal nociceptive neuronal excitability within a rat model of trigeminal pain, we will first introduce the trigeminal pain pathway and the general properties of nociceptive neurons. Within the trigeminal nervous system, pain signal transmission is generally classified into two distinct pathways: the lateral and the medial systems [23]. Experimental findings suggest that the lateral pain transmission pathway conveys precise details regarding pain intensity and location within the peripheral receptive field. In contrast, the medial pain transmission pathway is thought to relay information on the emotional component of pain, such as its perception as “pleasant or unpleasant,” to higher brain centers [26,28]. Nociceptive input from the orofacial area is transmitted by trigeminal ganglion (TG) neurons to the trigeminal spinal nucleus caudalis (SpVc)/upper cervical dorsal horn (C1–C2) [29,30]. Within this region, two types of neuron respond to noxious stimuli. Wide-dynamic-range (WDR) neurons transmit “pain level” perceptions to the central nervous system. Their firing frequency correlates with stimulus intensity, and they respond to both painful and non-painful inputs [23,29]. These neurons may also be involved in the development of hyperalgesia [23,29]. In contrast, nociceptive-specific neurons respond exclusively to noxious stimuli, potentially conveying location-specific information to higher brain centers [23,29]. From the SpVc/C1-2, two distinct pathways process different aspects of pain. The discriminative pathway projects to the primary and secondary somatosensory cortices via the medial ventral thalamic nucleus, while the affective pathway projects to the limbic amygdala, insular cortex, and anterior cingulate cortex via the parabrachial nucleus. This latter pathway is responsible for the emotional and comprehensive interpretation of pain [23,29,30].

## 4. Understanding the Peripheral and Central Transmission of Nociceptive Pain

**Figure 1:**
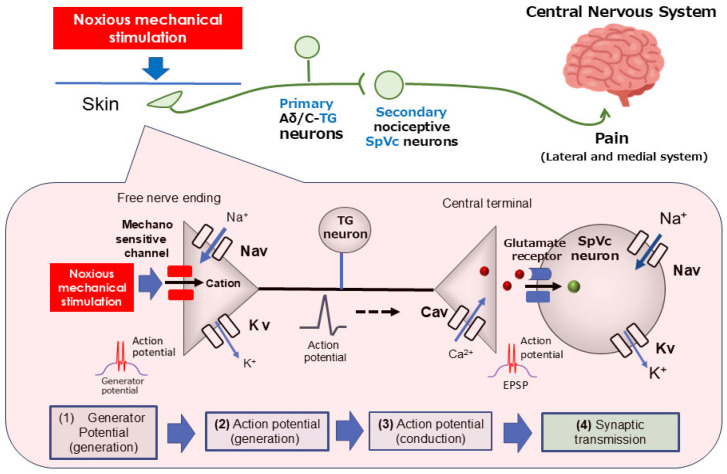
The transduction of trigeminal nociceptive signals is driven by a precise signaling cascade. This process begins when the noxious mechanical stimulation of tissue generates a potential in the peripheral terminals of trigeminal ganglion (TG) neurons. The resulting depolarization activates both Nav and Kv channels, producing action potentials. These signals propagate along the primary afferent trigeminal ganglion (TG) neurons, reaching the central terminal in nociceptive neurons located within the spinal trigeminal subnucleus caudalis (SpVc). The opening of presynaptic Cav channels triggers the release of neurotransmitters into the synaptic cleft. Upon binding to postsynaptic ionotropic glutamate receptors, these neurotransmitters elicit excitatory postsynaptic potentials (EPSPs). When the EPSP amplitude reaches the action potential threshold, a cascade of action potentials is sent to higher pain centers, ultimately causing the perception of pain. TG = trigeminal ganglion; Nav = voltage-gated sodium channel; Kv = voltage-gated potassium channel; Cav = voltage-gated calcium channel; SpVc = spinal trigeminal nucleus caudalis; EPSP = excitatory postsynaptic potential; ASIC = acid-sensing ion channel; TRPA1 = transient receptor potential ankyrin 1.

As illustrated in Figure 1, pain transmission involves primary sensory nerve fibers, including TG neurons, the thinly myelinated Aδ-fibers (known for slow conduction), and unmyelinated C-fibers [23,29,30]. Aδ-fibers swiftly carry sharp, intense, and localized pain signals. In contrast, C-fibers transmit diffuse, prolonged, and often dull pain sensations that are challenging to pinpoint [22,23]. TG neurons are characterized by their pseudo-bipolar cell morphology. Their central axonal projections establish chemical synapses with secondary neurons, while their peripheral ends serve as free nociceptive nerve terminals [23,29,30]. These nociceptors function as transducers, converting various noxious external energies (e.g., thermal, cold, mechanical, chemical) into electrical signals [22]. The general process for sensory information in primary afferent fibers unfolds in four crucial steps: (i) transduction, involving the conversion of external stimuli at the peripheral terminal; (ii) action potential generation and initiation; (iii) the propagation of these action potentials through neurons; and (iv) transmission, where the central terminal forms the presynaptic component of the initial synapse in the central nervous system’s sensory pathway [29,31].

When a painful mechanical stimulus stimulates the skin’s peripheral receptive field, candidate nociceptive channels like transient receptor potential ankyrin 1 (TRPA1) and acid-sensing ion channel (ASIC) become active. This activation initiates a depolarizing (generator) potential as cations flow into the cell through these ion channels [32,33,34]. The generator potential is a non-conductive, local analog signal whose amplitude grades with stimulus intensity, contrasting with the all-or-none conducting digital nature of an action potential. Consequently, generator potentials from primary sensory neuron nociceptors are called “trigger potentials” because they initiate action potentials. Noxious stimuli on free nerve endings can evoke potentials that surpass the action potential threshold. The depolarization phase is initiated by the activation of voltage-gated Na^+^ (Nav) channels, causing a sodium ion influx. This is followed by the repolarization phase, which involves potassium ion efflux through voltage-gated K^+^ (Kv) channels [29,35]. Nociceptive neurons possess both tetrodotoxin-sensitive (TTX-S) and TTX-resistant (TTX-R) Nav channels. Specifically, Aδ neurons contain both types, while C-neurons are predominantly equipped with TTX-R Nav channels [36]. An increase in noxious stimulus intensity raises the amplitude of the generator potential, which in turn leads to a higher firing frequency of subsequent action potentials [23,29]. These action potentials, generated at the free nerve terminals, propagate to the central axon ends via Nav and Kv channels distributed along the axons. Upon arrival at the central end, the action potential opens voltage-gated Ca^2+^ (Cav) channels, causing a Ca^2+^ influx. The resulting rise in intracellular Ca^2+^ concentration triggers the release of excitatory neurotransmitters, such as glutamate, into the synaptic cleft. This activates ionotropic glutamate receptors on secondary sensory neurons, allowing cation influx and generating an excitatory postsynaptic potential (EPSP). An action potential is initiated when the EPSP reaches a specific membrane potential threshold. The magnitude of the EPSP is believed to be proportional to the amount of transmitter released, with this heightened firing rate subsequently being interpreted by higher CNS regions as pain intensity [23,29].

## 5. Possible Mechanisms Underlying SCFAs’ Modulation of Pain

SCFAs are produced by the gut bacterial fermentation of dietary fibers. They are rapidly absorbed by the epithelium, where they generate ATP and provide energy for colonocytes [37]. After absorption, SCFAs are transported into the portal circulation and metabolized in hepatocytes [38]. As the gut’s primary metabolites, SCFAs are considered a key regulator of gut–brain communication [39]. These peripherally generated SCFAs can cross the BBB to enter the brain via monocarboxylate transporters [40], which are abundantly expressed in endothelial cells [41].

**Figure 2:**
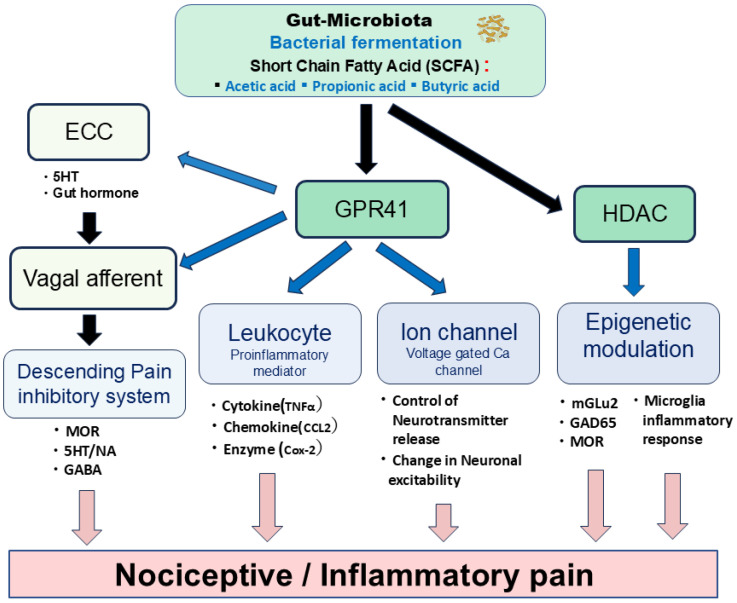
Schematic diagram showing potential mechanisms by which SCFAs modulate nociceptive and inflammatory pain. SCFAs could contribute to nociceptive and inflammatory pain modulation through the following mechanisms: (i) the histone deacetylase (HDAC)-mediated epigenetic mechanism; (ii) ionic channel modulation via the GPR41 mechanism; (iii) regulation of inflammatory mediators via the GPR41 mechanism; (iv) activation of vagal afferents via direct and indirect modulation. ECC = enterochromaffin cells; MOR = μ-opioid receptor; 5HT = serotonin; NA = noradrenaline; GABA = gamma-butylic acid; TNFα = tumor necrosis factorα; CCL2= chemokine C-C motif ligand 2. Cox-2 = cyclooxygenase-2; MGLU2 = metabotropic glutamate receptor 2; GAD65 = Glutamic acid decarboxylase 65.

Figure 2 depicts the potential mechanisms through which SCFAs might modulate both nociceptive and inflammatory pain. Specifically, SCFAs could contribute to pain modulation via epigenetic mechanisms involving the histone deacetylase (HDAC)-mediated control of neuronal excitability, ionic channel modulation facilitated by GPR41, the regulation of inflammatory mediators also through GPR41, and both the direct and indirect activation of vagal afferents.

Histone acetylation represents a prevalent epigenetic mechanism involved in gene expression regulation. Notably, sodium butyrate has been identified as a non-competitive HDAC inhibitor, exhibiting selective inhibitory effects on various Class I and IIa HDAC subtypes [42,43]. HDAC inhibition has been demonstrated to regulate chronic pain through several mechanisms. These include the attenuation of inflammatory responses in microglia after peripheral nerve injury [44,45], the modulation of glutamic acid decarboxylase 65 (GAD65), a GABA synthetic enzyme, in the brainstem nucleus raphe magnus [46], and the upregulation of metabotropic glutamate receptor 2 (mGlu2) and restoration of µ-opioid receptors in the spinal cord [47,48]. Consequently, SCFA-mediated HDAC modulation contributes to the attenuation of nociceptive signals in nociceptive/inflammatory pain.

GPR41 (FFAR3) functions as a Gi-coupled GPCR, whereas GPR43 (FFAR2) is a Gi/o- and Gq-dual-coupled GPCR [5]. GPR41/FFAR3 expression is observed in enterochromaffin cells (ECCs), sensory ganglion neurons, and brain tissues [5,49]. Given that SCFAs suppress N-type voltage-gated Ca (Cav) channel currents in isolated sympathetic ganglion neurons in vitro via GPR41 [50], it can be inferred that SCFAs modulate synaptic transmission and neuronal excitability, including that of sensory neurons [49]. Vinolo et al. [51] showed that SCFAs regulate several leukocyte functions, including cytokine (e.g., tumor necrosis factor α: TNFα) and chemokine (e.g., chemokine C-C motif ligand 2: CCL2) production, suggesting their therapeutic application for treating inflammatory responses-mediated inflammatory pain.

Additionally, SCFAs regulate the function of intestinal ECCs by acting on vagal afferents, leading to the release of serotonin or gut hormones that activate respective receptors on vagal afferent fibers [52,53]. Previous studies have demonstrated that activating vagal afferents stimulates the endogenous descending inhibitory system [54,55,56,57]. Therefore, these findings suggest that the SCFA-mediated activation of vagal afferents effectively modulates nociceptive inflammatory pain. Further studies are crucial to elucidate the underlying precise mechanisms.

## 6. Potential Modulatory Mechanisms of Nociceptive Pain by SCFAs

Recent findings indicate that propionic acid, an SCFA, suppresses N-type Cav channel currents in isolated sympathetic ganglion neurons in vitro via GPR41 [50]. N-type Cav channels are widely distributed in the central axon terminals of both peripheral and central nerves, playing a key role in synaptic transmission by releasing neurotransmitters [58]. Furthermore, Nohr et al. [49] reported that GPR41 expression extends beyond the autonomic nervous system to include TG neurons. These findings collectively propose that systemic SCFA administration alters nociceptive neuronal transmission in the SpVc through GPR41 signaling pathway-mediated inhibition of Cav channels in TG neuron nerve terminals. However, current knowledge is limited regarding the acute impact of SCFAs on nociceptive transmission under in vivo conditions.

In a recent study, Sashide and Takeda [59] explored whether systemic SCFA administration could reduce the excitability of SpVc WDR neurons. Their investigation revealed several key findings: (i) They observed a significant, dose-dependent inhibition of the mean SpVc WDR neuronal firing rate induced by the intravenous administration of an SCFA, propionic acid, in response to both non-noxious and noxious mechanical stimuli. (ii) This reduction in discharge frequency was reversible for both types of stimulus, manifesting within roughly 20 min. (iii) Notably, the inhibitory effect of SCFAs on SpVc WDR neuronal discharge frequency was significantly more pronounced for noxious stimuli than for non-noxious ones. (iv) The inhibitory action of propionic acid on SpVc firing frequency was abrogated by co-administering the GPR41 antagonist, (R)-(-)-3-hydroxybutyric acid. (v) Importantly, vehicle injection produced no significant impact on SpVc WDR neuronal activity across non-noxious or noxious mechanical stimuli. These results collectively indicate that acute intravenous propionic acid administration suppresses trigeminal nociceptive neuronal excitability in vivo. This effect is likely mediated by the activation of GPR41 signaling-mediated inhibition of Cav channel currents, presumably within primary sensory neurons.

**Figure 3:**
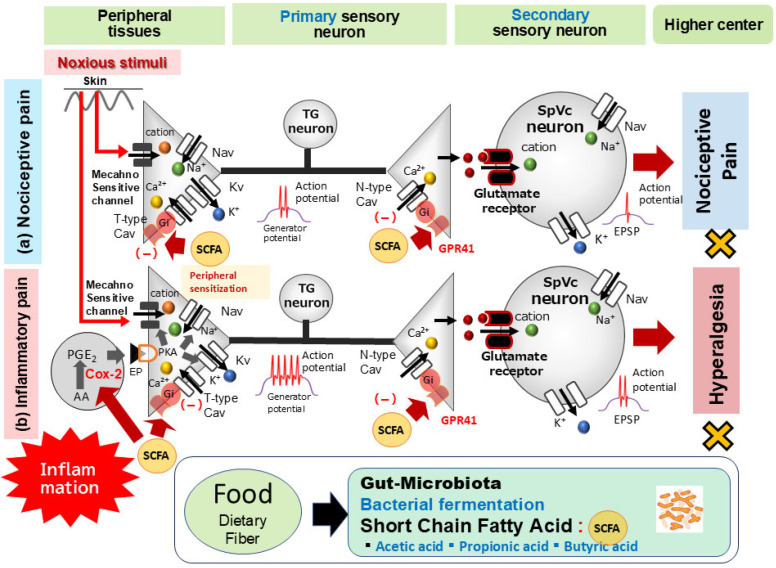
The possible mechanism underlying the systemic administration of short-chain fatty acids (SCFAs) inhibiting nociceptive and inflammatory pain. (a) *Nociceptive Pain*: When noxious mechanical stimulation affects the skin, mechanosensitive ion channels open, triggering generator potentials. This depolarization then opens voltage-gated Na^+^ (Nav) and K^+^ (Kv) channels, generating action potentials. These signals travel through primary afferent fibers to the trigeminal spinal nucleus caudalis (SpVc)’s central nociceptive neuron terminal. When an action potential reaches the central end of a nerve terminal, voltage-gated Ca^2+^ (Cav) channels open, leading to depolarization and a Ca^2+^ influx. The resulting rise in intracellular Ca^2+^ prompts the release of excitatory neurotransmitters, such as glutamate (Glu), from the presynaptic neuron into the synaptic cleft. Glu then activates ionotropic glutamate receptors on secondary sensory neurons, allowing cation influx and generating an excitatory postsynaptic potential (EPSP). An action potential is initiated when the EPSP reaches a specific membrane potential threshold. Intravenous administration of propionic acid, a short-chain fatty acid (SCFA) from the gut microbiota, suppresses the excitability of wide-dynamic range (WDR) neurons in the spinal trigeminal subnucleus caudalis (SpVc). This occurs because propionic acid activates G-protein coupled receptor 41 (GPR41) signaling, which inhibits presynaptic Cav channels in trigeminal ganglion (TG) neurons. This ultimately reduces the firing rate of action potentials in SpVc WDR neurons that propagate to higher pain centers. (b) *Inflammatory Pain*: In contrast, during inflammatory pain, peripheral inflammation causes inflammatory mediators like prostaglandin E_2_ (PGE2) to bind E-type prostanoid (EP) receptors. This binding activates protein kinase A (PKA) in nociceptive peripheral terminals, which in turn phosphorylates mechanosensitive (TRPA1, ASIC), Nav, and Kv channels. This action lowers the activation thresholds of transducer channels and enhances peripheral terminal membrane excitability, resulting in a high-frequency conduction of action potentials to the presynaptic central SpVc terminals. This prompts a large release of glutamate into the synaptic cleft, which binds to upregulated postsynaptic glutamate receptors, augmenting EPSPs and leading to a barrage of action potentials reaching higher pain centers, ultimately causing peripheral sensitization. As a systemic SCFA, butyrate mitigates mechanical inflammatory hyperalgesia associated with SpVc neuron hyperexcitability. Its mechanism involves inhibiting peripheral cyclooxygenase-2 (Cox-2) cascade signaling pathways and N-/T-type Cav channels, effectively normalizing SpVc neuronal hyperactivity.

Although existing knowledge, notably from Won et al. [50], has primarily associated SCFAs with N-type Cav channels, T-type Cav channels in primary afferents also play a critical role in the pain pathway. These T-type channels are essential for sustaining neuronal firing and seem to be involved in neurotransmitter release at spinal dorsal horn afferent terminals [60,61]. This increase in neuronal excitability consequently amplifies sensory transmission, resulting in intensified sensory processing, enhanced neuronal excitability, and the perception of pathological pain [62]. The seminal work by Gambeta et al. [62,63] further established T-type calcium channels as crucial regulators of neuronal function within the trigeminal system, profoundly impacting trigeminal pain. Considering these insights, the acute intravenous administration of SCFAs appears to reduce trigeminal nociceptive neuronal excitability. This effect is hypothesized to occur via T-type Cav channels, ultimately leading to the inhibition of Na^+^ channels and activation of K^+^ channels in TG neurons (Figure 3).

Vagal afferent fibers, situated within different layers of the gastrointestinal wall, including the mucosa, indirectly detect luminal signals from gut hormones and bacterial metabolites like SCFAs [64]. These fibers provide a crucial link between the gut microbiota and the brain, as their chemoreceptors are activated by gut hormones and SCFAs [65]. The SCFA receptor GPR41/FFAR3 is notably expressed in vagal neurons [49]. Previous studies have consistently shown that activating vagal afferents stimulates the endogenous descending inhibitory system [54,55,56]. This descending system can also be initiated by various physiological cues. For example, tooth-pulp electrical stimulation activates the jaw-opening reflex, generating neuronal activity in the spinal trigeminal nucleus oralis (SpVo) that subsequently inhibits vagal afferent conditioning stimulation [55]. Takeda et al. [56] further showed that physiological stimulation of vagal afferents, specifically through volume expansion, induced nociceptive transmission in SpVo neurons linked to the jaw-opening reflex, and that this inhibitory effect operated via the endogenous opioid system (involving the periaqueductal gray–nucleus raphe magnus–trigeminal pathway). Concurrently, other investigations reported that conditioning peripheral nerve stimulation suppresses nociceptive stimulation-evoked SpVc neuron activity through 5-HT3 receptor-mediated GABAergic inhibition [66,67]. These combined observations indicate that the low-pressure cardiopulmonary baroreceptor, whose afferents traverse the vagal nerve, inhibits trigeminal nociceptive transmission [56]. Given Goswami et al.’s [68] previous report that intraperitoneal SCFA injection activates vagal afferent neurons to suppress food intake, it is plausible that systemic SCFA injection inhibits nociceptive SpVc WDR neurons by triggering vagal afferents and, consequently, the descending inhibitory system. Consequently, these findings lead us to propose that propionic acid administration suppresses trigeminal nociceptive neuronal excitability not only through Cav channels, but also via activation of the vagal afferent-induced descending opioid inhibitory system. However, further studies are needed to elucidate this possibility.

## 7. Potential Modulatory Mechanisms of Inflammatory Pain by SCFAs

Animal models provide evidence for the therapeutic potential of butyrate, an SCFA, in treating pathological pain, specifically inflammatory and neuropathic conditions [4,12]. The analgesic actions of butyrate stem from diverse mechanisms, including epigenetic regulation via HDAC inhibition [69], the modulation of nuclear factor-kappa B (NF-κB) signaling [70], and direct interactions with the G-protein coupled receptors GPR41 and GPR43 [5,6]. While butyrate effectively attenuates neuropathic pain—potentially by decreasing inflammatory markers such as cyclooxygenase-2 (Cox-2), inducible nitric oxide synthase, cytokines [tumor necrosis factor α (TNFα), interleukin (IL)-2, IL-6 and IL-10], eicosanoids and chemokine [chemokine-C-C motif ligand 2 (CCL-2)] [12,51,71,72]—the precise neurophysiological mechanisms governing its modulation of nociceptive neuron hyperexcitability are still not fully understood.

Sekiguchi et al. [73] recently explored the potential of chronic resveratrol administration to suppress nociceptive neuron hyperexcitability in a rat model of CFA-induced inflammatory hyperalgesia. Our findings revealed a significant reduction in the withdrawal reflex threshold to von Frey hair mechanical stimulation in CFA-inflamed rats compared to naive rats; systemic resveratrol administration reversed this effect. Two days post-CFA injection, the inflammation group showed evidence of central sensitization: a decreased mechanical stimulus threshold (indicative of SpVc WDR neuron hyperexcitability), increased spontaneous and evoked discharge frequencies, and enlarged receptive fields. All of these indicators of central sensitization were normalized by resveratrol treatment. This evidence, combined with recent findings, informed our hypothesis. Ma et al. [12] demonstrated that resveratrol significantly reduced CFA-induced temporomandibular joint inflammation and, importantly, reversed the CFA-induced decline in butyrate levels and associated gut bacterial populations. Furthermore, Murakami et al. [74] reported that activating butyrate–GPR41 signaling by *Porphyromonas gingivalis* is pivotal in periodontitis pathogenesis, even in the absence of overt periodontal inflammatory pain. Considering this body of evidence, we hypothesized that butyrate administration would effectively mitigate inflammation-induced TG neuron hyperexcitability, thereby leading to the amelioration of trigeminal hyperalgesia.

A recent study by Sashide et al. [75] aimed to determine if systemically administered SCFA butyrate could mitigate inflammation-induced hyperexcitability of TG primary neurons linked to mechanical inflammatory hyperalgesia in vivo. Their investigation yielded several important results: (i) Consistent with prior reports, CFA-inflamed rats showed a significantly reduced escape threshold from orofacial mechanical stimulation compared to their naive counterparts. (ii) Chronic butyrate administration over four days effectively restored this lowered mechanical threshold in inflamed rats to levels seen in naive controls. (iii) A significant increase in mean edema thickness was observed in CFA-inflamed rats relative to naive rats. (iv) Butyrate administration for four days markedly reduced the mean edema thickness in inflamed rats, normalizing it to control levels. (v) Importantly, vehicle administration had no significant impact on either the escape threshold or edema thickness in CFA-inflamed rats on day 4. These findings collectively indicate that systemic butyrate administration alleviates peripheral sensitization during inflammatory states. Although the exact mechanism through which butyrate exerts its effects on inflammation-induced hyperalgesia has not yet been fully elucidated, multiple possibilities exist. Notably, butyrate is known to decrease PGE_2_ production by inhibiting COX-2 cascades [71]. Consequently, these observations suggest that daily butyrate administration mitigates inflammation-induced hyperalgesia in whisker pads by suppressing COX-2, leading to reduced PGE_2_ production and subsequent peripheral sensitization. Beyond its impact on mechanical thresholds, a study by Sashide et al. [75] revealed that systemic butyrate administration restored the decreased mean mechanical stimulation threshold in inflamed rats. Notably, butyrate treatment normalized the mean discharge frequencies in trigeminal ganglion (TG) neurons—evoked by both non-noxious and noxious mechanical stimuli—bringing them back to control levels. A well-established mechanism shows that during peripheral inflammation, the pro-inflammatory mediator PGE2 binds to G-protein-coupled E-type prostanoid (EP) receptors. This binding activates protein kinase A (PKA) in nociceptive peripheral terminals [29]. PKA proceeds to phosphorylate mechanosensitive TRPA1, Nav, and Kv channels. This action reduces the activation threshold of TRPA1 channels and enhances membrane excitability in the peripheral terminals of TG neurons. The consequence is a higher frequency of nerve impulses being transmitted to the presynaptic central terminals of the SpVc. These findings suggest that systemic butyrate may regulate inflammation-induced peripheral sensitization and TG neuronal hypersensitivity at peripheral nerve terminals. This observation is consistent with prior in vitro studies indicating that butyrate modulates neuronal activity via Cav channel regulation [50] (Figure 3).

In a related study, Sashide et al. [75] showed that butyrate could reverse the heightened mean spontaneous discharge frequency of TG neurons observed after inflammation. The origin of this ongoing activity in central neurons, which is responsible for relaying sensory information, holds significant clinical importance, as it may be a determinant of post-traumatic injury and chronic pain severity [76]. A more recent study [77] provided evidence that the ongoing activity of SpVc WDR neurons is driven by peripheral input, given that a microinjection of lidocaine into the trigeminal ganglia markedly reduced this activity. Additionally, butyrate has been shown to inhibit the sodium–potassium–chloride cotransporter in the rat colon, leading to elevated intracellular chloride ion concentrations [78,79]. Based on these findings, we can speculate that this effect in neurons might trigger hyperpolarization by increasing intracellular chloride ion concentrations and promoting potassium ion efflux. Such an action would consequently decrease the frequency of action potential discharges in all neurons, including those involved in nociception. In conjunction with our findings, this indicates that butyrate reduces the heightened spontaneous discharge activity of TG neurons that innervate the whisker pad. This hyperactivity is a result of sensitization at the periphery and/or within the trigeminal ganglion itself.

## 8. Functional Role of SCFAs in Pain Modulation and Future Direction

It is known that SCFAs originate mainly from the bacterial fermentation of carbohydrates and protein, resulting in plasma concentrations ranging from 0.1 to 10 mM [80,81]. Building on this, Frost et al. [82] reported that systemic SCFA administration reduced food intake by crossing the blood–brain barrier and directly activating hypothalamic neurons. Our recent work [58] further revealed that acute intravenous administration of physiological SCFA concentration significantly inhibited trigeminal nociceptive neuronal excitability through GPR41 signaling-mediated Cav channel inhibition. Collectively, these results propose that the systemic administration of gut microbiota-derived SCFAs modulates trigeminal nociception. This positions SCFAs as potential intravenous analgesic agents for trigeminal pain relief, encompassing conditions like orofacial clinical pain and visceral pain, thereby offering a new therapeutic avenue. The swift anesthetic effect and sedative properties achievable with short-term intravenous SCFA administration present a promising advantage for minimizing side effects in clinical procedures where anesthesia is essential (e.g., endoscopy).

Nevertheless, additional research is required to fully substantiate this hypothesis. Consistent with our previous reports on the pain-alleviating potential of natural products, especially phytochemicals, as discussed in previous review papers [83], we have now electrophysiologically confirmed that SCFAs can mitigate inflammatory nociceptive hypersensitivity through mechanisms including COX-2 inhibition [75]. This highlights the potential for gut bacterial-derived SCFAs from dietary fiber fermentation to serve as substitutes for non-steroidal anti-inflammatory drugs (NSAIDs).

Gene expression within nociceptive pathways is well known to play a crucial role in inducing and maintaining persistent pain, including inflammatory pain [84,85,86]. Sodium butyrate has been reported as a non-competitive inhibitor of HDAC that selectively inhibits multiple subtypes of Class I and IIa HDACs [42,43]. Given that HDAC IIa inhibitors can inhibit complete CFA-induced hyperalgesia, previous studies have suggested a key role for HDAC IIa in inflammatory pain [87]. This collective evidence indicates that epigenetic regulation within pain pathways contributes to the development of persistent pain and influences analgesic effects. Therefore, it can be tentatively proposed that butyrate-mediated HDAC inhibition likely accounts for its pain-attenuating effect in trigeminal pathological pain, including persistent inflammatory pain. These findings demonstrate that SCFA administration alleviates CFA-induced inflammatory pain, including trigeminal mechanical hyperalgesia, suggesting a significant role for SCFAs in pain relief. Our previous study [88] investigated the efficacy of resveratrol in a rat model of orthodontic ectopic hyperalgesia. We found that resveratrol effectively reduced this hyperalgesia by inhibiting the hyperexcitability of pain-transmitting SpVc WDR neurons, all without compromising tooth movement. Given these results, further studies are warranted to investigate the potential association between gut microbiota-derived SCFAs and pathological pain conditions, such as orthodontic ectopic pain.

A previous study by Tang et al. [15] established a link between chronic antibiotic use and the chronicity of NTG-induced acute migraine-like pain, demonstrating that gut microbiota disturbance is the causal factor. Importantly, they showed that restoring the microbiome with probiotics prevents this chronicity. Tang et al. [15] further discovered that this dysbiosis enhances migraine-like pain through TNFα upregulation in the SpVC. As TNFα is a pro-inflammatory cytokine, it is a key player in chronic pain development. This role is supported by findings that TNFα enhances the phosphorylation and trafficking of the α-amino-3-hydroxy-5-methyl-4-isoxazolepropionic acid (AMPA) receptor GluA1 in spinal dorsal horn neurons, thereby contributing to inflammatory pain [89,90]. Based on this evidence, we conclude that gut microbiota dysbiosis is critical for migraine-like pain chronicity. We hypothesize that re-establishing a healthy gut microbiome is an effective therapeutic strategy, specifically through active substances like SCFAs, which are generated by a healthy intestinal microbiome and may be beneficial in managing migraines.

The influence of microbial metabolites on chronic pain is a growing area of research. Pane et al. [91] recently reported that microbial metabolites, such as SCFAs, can modulate the blood–brain barrier and nociceptive processing, providing potential therapeutic avenues for chronic pain. This finding is consistent with a previous report from the same group [92], which established a gut–brain axis involving SCFAs, neuroinflammation, and nociceptive behavior, thus supporting the broader concept of microbiota-pain interactions.

GPR109A (HCAR2) is an important SCFA-sensing receptor involved in chronic pain, in addition to GPR41 and GPR43 [93,94]. This Gi-coupled receptor is activated by butyrate and β-hydroxybutyrate and is found in immune cells, glial cells, and neurons. Boccella et al. [93] showed that GPR109A activation reduces neuropathic pain by modulating microglial activity and cytokine release in the spinal cord, suggesting anti-inflammatory and analgesic effects. This is supported by findings that GPR109A activation suppresses NF-κB signaling and pro-inflammatory cytokines such as TNF-α and IL-1β [94], which helps inhibit central sensitization and restore neuroimmune balance. These findings suggest that GPR109A is a promising therapeutic target for chronic and neuropathic pain.

Our current understanding indicates that the modifying function of SCFAs on excitatory nerve conduction involved in pain transmission, and the associated excitatory elements, has only been reported for voltage-gated Ca channels. Information concerning other voltage-gated ion channels, as well as neurotransmitters and their receptors, remains unreported. Consequently, future in vitro and in vivo experimental investigations into these areas are essential.

For the eventual clinical implementation of the research findings presented herein, additional foundational research focused on clinical translation will be indispensable. Future investigations should employ germ-free animal models to further elucidate the molecular targets of SCFAs. This would entail electrophysiological analysis of transmission system modulation during pain perception and transmission, alongside the verification of pain hypersensitivity in conditions of reduced SCFA production.

Finally, to better classify pain that arises without clear signs of tissue or nerve damage, the International Association for the Study of Pain recently established the term nociplastic pain. This new classification includes conditions that were once referred to as psychogenic or non-organic pain [95]. The pathophysiology of nociplastic pain is primarily linked to central nervous system dysfunction, particularly central sensitization. Furthermore, the emotional dimension of pain, controlled by the medial pain pathway, is considered a significant factor [96]. Given the medial system’s influence on the emotional components of pain and its role in mechanisms like central sensitization, it is highly probable that this system is involved in the multifaceted pathology of nociplastic pain. Previous research has shown that SCFAs can reduce peripheral sensitization, which is a key contributor to central sensitization in the lateral pain pathway. Therefore, future studies should investigate the clinical applicability of SCFAs in alleviating nociplastic pain, specifically by targeting these interconnected pathways.

## 9. Concluding Remarks

The gut microbiota orchestrates energy metabolism and central nervous system (CNS) functions through a complex interplay of humoral factors (metabolites, neurotransmitters, cytokines, and hormones) and neural pathways such as the vagus nerve. Among these, short-chain fatty acids (SCFAs)—including acetic, propionic, and butyric acids—are produced by the gut microbiota via anaerobic fermentation of dietary fibers. A growing body of evidence highlighting their beneficial effects on various pain conditions makes SCFAs of significant interest.

Based on prior in vitro data, we recently moved to in vivo animal experiments. Using a neurophysiological approach, we assessed the pain-relieving potential of SCFAs. As summarized in Figure 3, our key findings demonstrate two distinct mechanisms: (i) acute nociceptive pain attenuation, in which intravenous SCFA administration acutely lowered trigeminal nociceptive neuronal excitability. This effect appears to be mediated by T-type Cav channels, which inhibit Na^+^ channels and activate K^+^ channels in TG neurons; and (ii) chronic pain relief via anti-inflammatory action, in which daily butyrate administration successfully mitigated inflammation-induced hyperalgesia. This process involved COX-2 suppression, leading to reduced PGE_2_ production, ameliorated peripheral sensitization, and central terminal Cav channel inhibition. This highlights the potential for gut bacterial-derived SCFAs from dietary fiber fermentation to serve as substitutes for NSAIDs.

This review proposes that SCFAs represent a significant advancement in alleviating both nociceptive and pathological pain. Their considerable potential as a non-opioid therapeutic target for pathological pain warrants further investigation. Comprehensive mechanistic studies are crucial to fully characterize how SCFAs modulate nociceptive transmission.

## Data Availability

Not applicable.

## Abbreviations

The following abbreviations are used in this manuscript:

CNS	Central nervous system
SCFA	Short-chain fatty acid
GPR	G-protein-coupled receptor
FFAR	Free fatty acid receptor
HCAR	Hydroxy-carboxylic acid receptor
SPF	Specific pathogen-free
GF	Germ-free
NTG	Nitroglycerin
FMT	Fecal microbiota transplantation
IBS	Irritable bowel syndrome
TG	Trigeminal ganglion
C1-C2	Upper cervical dorsal horn
SpVc	Trigeminal spinal nucleus caudalis
SpVc	Trigeminal spinal nucleus oralis
WDR	Wide-dynamic range
Nav	Voltage-gated Na channel
Kv	Voltage-gated K channel
Cav	Voltage-gated Ca channel
EPSP	Excitatory post synaptic potential
ASIC	Acid sensing channel
TRPA1	Transient receptor ankyrin 1
TTX	Tetrodotoxin
HDAC	Histone deacetylase
MOR	μ-opioid receptor
5HT	Serotonin
NA	Noradrenaline
GABA	γ-aminobutylic acid
TNFα	Tumor necrosis factorα
CCL2	Chemokine c-c ligand 2
IL	Interleukin
CFA	Complete Freund’s adjuvant
COX-2	Cyclooxygenase 2
MGLU2	Metabotropic glutamate receptor 2
GAD65	Glutamic acid decarboxylase 65
PKA	Protein kinase A
PKC	Protein Kinase C
EP	E-type prostanoid
PGE_2_	Prostaglandin E_2_
Glu	Glutamate
NF-κB	Nuclear factor-kappa B
BBB	Blood–brain barrier
